# Conjunctival sac flora and drug susceptibility analysis in normal children in East China

**DOI:** 10.1186/s12886-023-02995-1

**Published:** 2023-06-02

**Authors:** Mingming Jiang, Jing Zhang, Xiaomei Wan, Yichao Ding, Feijia Xie

**Affiliations:** 1 Eye Institute of Shandong First Medical University, Qingdao Eye Hospital of Shandong First Medical University, No.5 Yan’erdao Road, Shinan District, Qingdao, Shandong China; 2 State Key Laboratory Cultivation Base.Shandong Provincial Key Laboratory of Ophthalmology, Qingdao, Shandong China; 3 School of Ophthalmology, Shandong First Medical University, Qingdao, Shandong China

**Keywords:** Conjunctival sac flora, Drug susceptibility, Normal children

## Abstract

**Purpose:**

To investigate the distribution characteristics of conjunctival sac flora and assess the susceptibility of commonly used topical antimicrobial agents in normal children under the age of 18 in East China.

**Methods:**

In 2019, a study was conducted at Qingdao Eye Hospital of Shandong First Medical University to analyze the microorganism cultures of conjunctival sac in 1258 normal children (2516 eyes; average age, 6.21 ± 3.78 years) in East China. Exclusion criteria included children with ocular surface diseases and those who had used any topical antimicrobial agents recently. The microorganism species in the conjunctival sac were analyzed using the M-38A protocol (microdilution method; investigators read the minimum inhibitory concentration [MIC] values) by the Clinical and Laboratory Standards Institute to determine drug susceptibility.

**Results:**

The incidence of conjunctival sac microorganism in children was 32.87% (827/2516), a total of 541 cases (male 293, female 248). Children with conjunctival sac flora in a single eye were 255 and in both eyes were 286 (no statistical difference, *P* > 0.05). The concordance rate of children with binocular conjunctival sac flora was 32.16% (174/541; male 84, female 90). A total of 42 species of bacteria were detected. Children with Gram-positive cocci accounted for the highest proportion, 91.54% (757/827). The top three bacteria with the highest detection rates were *Staphylococcus epidermidis* (*S. epidermidis*; 52.12%), *Streptococcus* (12.09%), and *Staphylococcus aureus* (*S. aureus*; 10.76%). *Streptococcus mitis* (5.20%) accounted for the highest proportion of *Streptococcus*.*S. epidermidis* had the highest proportion in all age groups and was positively correlated with age (*r* = 0.89, *P* = 0.03). Before six years of age, the streptococcal proportion(*mainly S. mitis*) was greater than that of Staphylococcus aureus. The drug susceptibility analysis showed that *S. epidermidis* was most sensitive to gatifloxacin (98.61%), while it had the highest resistance rate to erythrocin (87.94%). *S. aureus* had the highest susceptibility to moxifloxacin (100%). *Streptococcus* was most sensitive to moxifloxacin (96.97%) and had the highest resistance rate to tobramycin (92.93%).

**Conclusions:**

Conjunctival sac flora in children was dominated by Gram-positive cocci, mainly *S. epidermidis*, *S. aureus*, and *Streptococcus*. *S. epidermidis* increased with age; the proportion of *Streptococcus* was higher than *S. aureus* among children aged 0–6 years. The typical conjunctiva sac flora was generally sensitive to quinolones, such as moxifloxacin and gatifloxacin; *Streptococcus* displayed high resistance to tobramycin antibiotics; and the female children had higher resistance to tobramycin than the male children.

## Introduction

The conjunctival sac is an area in direct contact with the external environment, located between the bulbar and palpebral conjunctiva. It is inhabited by a variety of aerobic and anaerobic flora that coexist to maintain the ocular surface microenvironment. However, the normal flora in the conjunctival sac is a potential pathogen, as it can cause severe eye infections under certain circumstances, such as surgery, trauma, or weakened immunity [1]. Diagnosing eye infections in children and infants is challenging, and ensuring compliance with local medication can be difficult. Additionally, intraocular surgeries and postoperative care in children can be complicated and are associated with a higher risk of complications. Therefore, understanding the distribution characteristics of conjunctival sac flora, drug susceptibility, and timely use of antibiotics can help prevent serious eye infections and guide perioperative medication in children. While there have been numerous studies on conjunctival sac flora in normal adults [2–4], research on children is comparatively scarce. Hence, the objective of this study was to culture conjunctival sac microorganisms from a large sample of 1258 normal children aged 0–18 years and to analyze the drug susceptibility of commonly isolated strains.

## Research object and methods

### Research object

Microorganism cultures of conjunctival sac were analyzed for 1258 children (male 643, female 615, *P* > 0.05; age, 6.21 ± 3.78 years) at Qingdao Eye Hospital of Shandong First Medical University in 2019. They visited the hospital for eye examination and refraction, and did not exhibit any clinical signs of ocular infection, such as conjunctival congestion or edema. Children with eye infections and other infectious symptoms were excluded from the study, as were children who had used antibiotics locally or systemically to control infection in the past month. Informed consent was obtained from the families of all children before the examination, and they were informed about the purpose and process of the study.

### Methods

#### Conjunctival sac sampling

Operators with clean hands or sterile inspection gloves cleaned the eyelids and surrounding skin with sterile normal saline. Participants were instructed to look up while the operator used their index finger to press down on the upper eyelid and their thumb to press down on the lower eyelid skin, fully exposing the conjunctival sac. Next, sterile artificial fiber swabs (3–5 laps) were rolled in the conjunctival sac across the inferior fornix of the bulbar conjunctiva for 3–5 s. The swabs with the specimens were then dipped into a sterile enrichment broth medium after breaking the plastic rod of the swab (2 cm shorter than the tube) and cultured at 37 °C as soon as possible. During the sampling process, the operators took special care to avoid touching the eyelid skin and eyelashes. Additionally, they made sure that the children did not blink during the sample collection. All the sampling procedures were performed by specialists to ensure accuracy and consistency.

#### Bacterial culture and drug susceptibility test

The collected specimens were inoculated, cultured, identified for strains, and tested for drug susceptibility at Qingdao Eye Hospital of Shandong First Medicine University. In brief, specimens were inoculated onto blood and chocolate plates and cultured in a 5% CO_2_ environment at 35 ± 2 °C for 24 h. The cultures were smeared and stained to identify the bacteria with a microscope, while the bacterial strains were identified using the Micro Scan Autoscan-4 system (Siemens Healthcare, Germany).

An antimicrobial susceptibility test was performed using the Automatic TDR-200B Bacteria and the antibiotics susceptibility analyzer (Jinyang Science & Technology, Beijing, China) according to the Clinical and Laboratory Standards Institute (CLSI) protocols [5]. Additionally, the broth microdilution method was used to determine the bacterial strain’s susceptibility to antibiotics. *Staphylococcus aureus* (ATCC29213), *Streptococcus pneumoniae* (ATCC49619), *Haemophilus influenzae* (ATCC49247), *Pseudomonas aeruginosa* (ATCC27853), *Escherichia coli* (ATCC25922), and *Enterococcus* (ATCC29212) were used as the quality control strains. The procedures for developing and analyzing the conjunctival sac flora species and their drug susceptibility were conducted according to the M-38A protocol (microdilution method) of the Clinical and Laboratory Standards Institute (CLSI), and the investigators read the minimum inhibitory concentration (MIC) values.

#### Statistical methods

SPSS 13.0 (IBM) was used for statistical analyses. The normality of continuous variables was assessed using the single-sample Kolmogorov–Smirnov test. Normally distributed variables were presented as mean ± standard deviation and were compared using two independent sample t-tests. Pearson correlation analysis was used for correlation analysis. A *p*-value of < 0.05 was considered statistically significant.

## Results

### Distribution characteristics of conjunctival sac flora

#### Basic information

A total number of 541 children (827 eyes) were found with a positive culture of conjunctival sac flora (32.87%); 293 males (54.15%) and 248 females (45.84%), the difference was not statistically significant (*P* > 0.05). There were 255 children (47.13%) with a positive monocular conjunctival sac culture (147 males and 118 females); the gender difference was not statistically significant (*P* > 0.05). There were 285 (52.86%) positive binocular conjunctival sac cultures observed (146 males and 140 females); the gender difference was not statistically significant (*P* > 0.05). Additionally, 174 chindren (32.16%) with consistent binocular conjunctiva sac flora were reported (84 males and 90 males), and the difference was not statistically significant (*P* > 0.05).

#### Microbial flora distribution

**Fig. 1:**
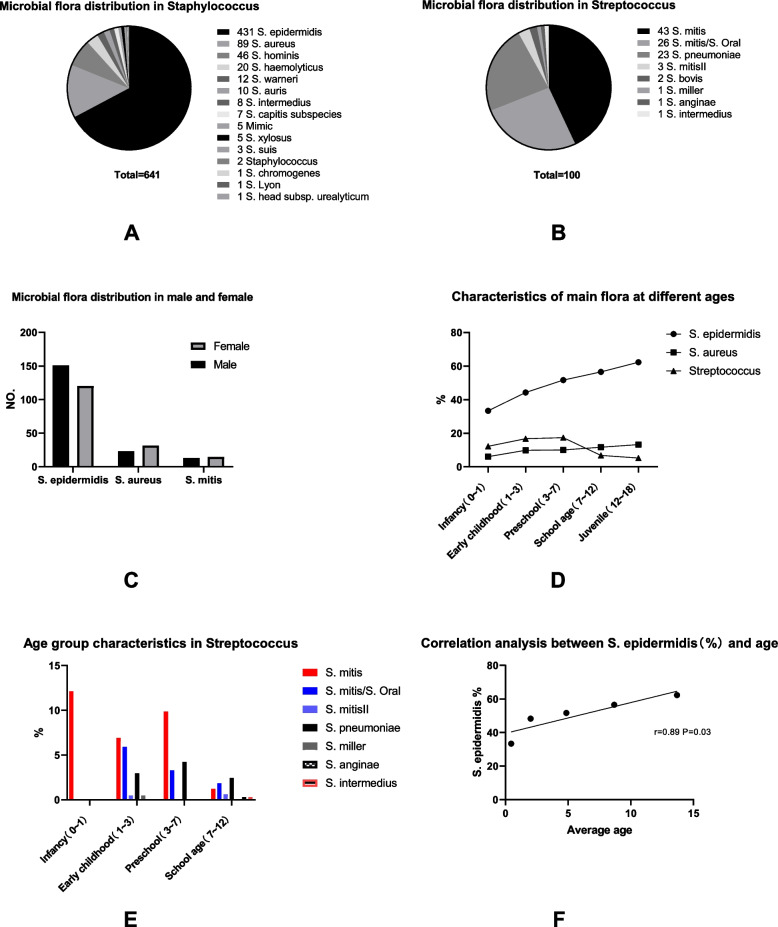
**A** Microbial flora distribution in Staphylococcus. **B** Microbial flora distribution in Streptococcus. **C** Microbial flora distribution in male and female,Only the top three strains are listed. **D** Characteristics of main flora at different ages. **E** Age group characteristics in Streptococcus. **F** Correlation analysis between S. epidermidis(%) and age

42 bacterial species were detected among 827 samples: 91.54% Gram-positive cocci (757 cases), 3.99% Gram-positive bacillus (33 cases), 2.06% Gram-negative bacillus, and 2.42% fungi (Table 1). *Staphylococcus* (77.51%) was the highest observed microbial flora; *S. epidermidis* (52.2%) and *S. aureus* (10.76%) were identified as the predominant staphylococcal species (Fig. 1A). The second was *Streptococcus* (12.09%), of which* S*. *mitis* (5.20%) accounted for the highest proportion (Fig. 1B).

**Table 1:** Microbial flora distribution in normal children

	Microorganisms	NO	Percentage %
Gram-positive bacillus		33	3.99
	Corynebacterium	22	2.66
	Bacillus	10	1.21
	Rhodococcus	1	0.12
Gram-positive cocci		757	91.54
	Staphylococcus	641	77.51
	Streptococcus	100	12.09
	Micrococcus	6	0.73
	Enterococcus	5	0.6
	Aerococcus	4	0.48
	Geminicoccus	1	0.12
Gram-negative bacillus		17	2.06
	Acinetobacter	6	0.73
	Escherichia	3	0.36
	Monas	3	0.36
	Shewanella	3	0.36
	Morganella	1	0.12
	Yersinia	1	0.12
Fungi		20	2.42
	Microsporum	20	2.42
Total		827	100

#### Positive conjunctival sac flora in single and both eyes

A total of 32 bacterial species were detected in children with positive conjunctival sac flora in a single eye. *S. epidermidis* (54.90%), *S. aureus* (9.41%), *S. mitis* (3.14%), and *S. pneumoniae* (2.75%) were the most common floras. Children with positive conjunctival sac flora in both eyes reported 34 bacterial species, *S. epidermidis* (51.92%), *S. aureus* (11.19%), *S. mitis* (6.12%), and *S. pneumoniae* (2.87%). There was no statistically significant difference in the number of *S. epidermidis,S. aureus* and *S. pneumoniae* detected between the two groups (*P* > 0.05). Howere,the prevalence of *Streptococcus* was found to be higher in children with positive conjunctival sac flora in both eyes compared to those with positive flora in a single eye.

#### Gender characteristics

Thirty-five bacterial species were detected in male children; *S. epidermidis* (55.66%), *S. aureus* (8.60%), and *S. mitis* (4.52%) accounted for a higher proportion. There were 33 bacterial species detected in female children, and *S. epidermidis* (49.61%), *S. aureus* (12.99%), and *S. mitis* (5.97%) were quite common. There were no significant differences in the number of bacteria detected between different genders (*P* > 0.05)(Fig. 1C).

#### Age group characteristics

The proportion of *S. epidermitis* was the highest in all age groups and increased gradually with age(Fig. 1D). However, in children aged 0–6 (infancy, early childhood, and preschool), *Streptococcus(mainly S. mitis)* accounted for a higher proportion than *S. aureus*. Over the age of 6, the proportion of *Streptococcus* decreased and was lower than that of *S. aureus*, among which *S. pneumoniae* became the main strain (Figs. 1D and E).

#### Children with consistent conjunctival sac flora in both eyes

One hundred seventy-four chindren (32.16%) were presented with consistent binocular conjunctiva sac flora.The average age was 6.16 years. *S. epidermidis* (63.49%), *S. aureus* (11.49%), and *S. mitis* (6.90%) were the main floras.

#### Correlation analysis between *S. epidermidis*(%) and age

Correlation analysis identified a significant association between S. epidermidis and age(*r* = 0.89,*P* =  = 0.03)(Fig. 1F).

### Drug susceptibility analysis of bacterial flora

#### Bacterial flora

*S. epidermidis* was sensitive to quinolones, especially gatifloxacin (98.61%) and moxifloxacin (97.22%), and their resistance rate to erythromycin was the highest (87.94%). *S. aureus* was most sensitive to moxifloxacin and gatifloxacin, whereas *Streptococcus* was most sensitive to moxifloxacin (96.97%) with the highest resistance rate to tobramycin (92.93%) (Table 2).

**Table 2:** Drug susceptibility analysis of bacterial flora in children

	**Moxifloxacin**		**Levofloxacin**		**Ciprofloxacin**		**Gatifloxacin**		**Ofloxacin**		**Fusidic Acid**		**Tobramycin**		**Erythromycin**	
	**NO**	**%**	**NO**	**%**	**NO**	**%**	**NO**	**%**	**NO**	**%**	**NO**	**%**	**NO**	**%**	**NO**	**%**
**Staphylococcus epidermidis (431)**																
** S**	419	97.22	318	73.78	285	66.13	425	98.61	306	71.00	288	66.82	330	76.57	46	10.67
** I**	6	1.39	55	12.76	6	1.39	1	0.23	23	5.34	6	1.39	5	1.16	6	1.39
** R**	6	1.39	58	13.46	140	32.48	5	1.16	102	23.67	137	31.79	96	22.27	379	87.94
**Staphylococcus aureus (89)**																
** S**	89	100.0	86	96.63	83	93.26	89	100.0	88	98.88	40	44.94	43	48.31	22	24.72
** I**	0	0.00	3	3.37	2	2.25	0	0.00	1	1.12	2	2.25	0	0.00	2	2.25
** R**	0	0.00	0	0.00	4	4.49	0	0.00	0	0.00	47	52.81	46	51.69	65	73.03
**Streptococcus (99)**																
** S**	96	96.97	77	77.78	66	66.67	94	94.95	70	70.71	16	16.16	7	7.07	34	34.34
** I**	0	0.00	2	2.02	6	6.06	0	0.00	8	8.08	2	2.02	0	0.00	4	4.04
** R**	3	3.03	20	20.20	27	27.27	5	5.05	21	21.21	81	81.82	92	92.93	61	61.62

*S* Susceptible, *I* Intermediate, *R* Resistant

#### Gender analysis

The drug susceptibility trends were almost similar for both genders; *Staphylococcus* and *Streptococcus* were both sensitive to quinolones, particularly gatifloxacin and moxifloxacin. However, the *Streptococcus* resistance rate to tobramycin was higher in female children (Tables 3 and 4).

**Table 3:** Drug susceptibility analysis of bacterial flora in male children

	**Moxifloxacin**		**Levofloxacin**		**Ciprofloxacin**		**Gatifloxacin**		**Ofloxacin**		**Fusidic Acid**		**Tobramycin**		**Erythromycin **	
	**NO.**	**%**	**NO.**	**%**	**NO**	**%**	**NO**	**%**	**NO**	**%**	**NO**	**%**	**NO**	**%**	**NO**	**%**
**Staphylococcus epidermidis （244）**																
** S**	237	97.13	177	72.54	160	65.57	241	98.77	169	69.26	173	70.90	187	76.64	25	10.25
** I**	4	1.64	32	13.11	2	0.82	1	0.41	14	5.74	1	0.41	2	0.82	3	1.23
** R**	3	1.23	35	14.34	82	33.61	2	0.82	61	25.00	70	28.69	55	22.54	216	88.52
**Staphylococcus aureus (38)**																
** S**	38	100.00	37	97.37	36	94.74	38	100.00	38	100.00	18	47.37	20	52.63	10	26.32
** I**	0	0.00	1	2.63	1	2.63	0	0.00	0	0.00	0	0.00	0	0.00	0	0.00
** R**	0	0.00	0	0.00	1	2.63	0	0.00	0	0.00	20	52.63	18	47.37	28	73.68
**Streptococcus （48）**																
** S**	47	97.92	43	89.58	37	77.08	47	97.92	41	85.42	11	22.92	4	8.33	18	37.50
** I**	0	0.00	0	0.00	2	4.17	0	0.00	1	2.08	2	4.17	0	0.00	1	2.08
** R**	1	2.08	5	10.42	9	18.75	1	2.08	6	12.50	35	72.92	44	91.67	29	60.42

*S* Susceptible, *I* Intermediate, *R* Resistant

**Table 4:** Drug susceptibility analysis of bacterial flora in female children

	**Moxifloxacin**		**Levofloxacin**		**Ciprofloxacin**		**Gatifloxacin**		**Ofloxacin**		**Fusidic Acid**		**Tobramycin**		**Erythromycin **	
	**NO.**	**%**	**NO.**	**%**	**NO**	**%**	**NO**	**%**	**NO**	**%**	**NO**	**%**	**NO**	**%**	**NO**	**%**
**Staphylococcus epidermidis （191）**																
** S**	188	98.43	150	78.53	133	69.63	189	98.95	145	75.92	121	63.35	147	76.96	28	14.66
** I**	1	0.52	23	12.04	4	2.09	0	0.00	9	4.71	5	2.62	3	1.57	3	1.57
** R**	2	1.05	18	9.42	54	28.27	2	1.05	37	19.37	65	34.03	41	21.47	160	83.77
**Staphylococcus aureus (51)**																
** S**	51	100.00	49	96.08	47	92.16	51	100.00	50	98.04	22	43.14	23	45.10	12	23.53
** I**	0	0.00	2	3.92	1	1.96	0	0.00	1	1.96	2	3.92	0	0.00	2	3.92
** R**	0	0.00	0	0.00	3	5.88	0	0.00	0	0.00	27	52.94	28	54.90	37	72.55
**Streptococcus （46）**																
** S**	45	97.83	30	65.22	27	58.70	43	93.48	26	56.52	5	10.87	2	4.35	14	30.43
** I**	0	0.00	2	4.35	3	6.52	0	0.00	6	13.04	0	0.00	0	0.00	3	6.52
** R**	1	2.17	14	30.43	16	34.78	3	6.52	14	30.43	41	89.13	44	95.65	29	63.04

*S* Susceptible, *I* Intermediate, *R* Resistant

## Discussion

*Staphylococcus* and *Propionibacterium* were the predominant conjunctival sac floras in normal adults; *Corynebacterium* and *Streptococcus* were secondary, while other bacterial species were relatively lesser [6]. Previous studies have reported that the normal flora of the conjunctival sac is established during infancy [7] and that the structural composition of this flora remains constant throughout adulthood [8]. This study observed that *S. epidermidis* (52.12%), *Streptococcus* (12.79%), and *S. aureus* (10.76%) were the significant microbial floras in children, which was consistent with earlier studies [4, 9]. However, our study reported the proportion of *S. epidermidis* in children was much lower than that in adults. The conjunctival sac flora analysis in different age groups showed that *S. epidermidis* had the highest proportion in all age groups; the proportion increased gradually with age, similar to earlier research results [4, 10]. The results suggest that while the conjunctival sac flora is established during childhood, the proportion and distribution of the flora are still distinct from those of adults.

Earlier studies showed that *Staphylococcus* (37.4%) was the main conjunctival sac flora in healthy children, followed by *Corynebacterium* (30%) and *S. pneumoniae* (21.4%) [11]; the lower proportion of *Staphylococcus* could be attributed to the age of the object. Our study sample had a wider age range compared to Ke et al.’s study [11], which only included children aged 0 to 6 years. Additionally, our study found a negative correlation between age and the proportion of *Staphylococcus*, indicating that the younger the age, the lower the proportion of *Staphylococcus*. As age increased, the proportion of *S. epidermidis* gradually increased, approaching the levels observed in adults.

In addition to *Staphylococcus*, it was found that *Streptococcus* also had a high detection rate (12.9%) in the conjunctival sac of children, including *S. pneumoniae*, *S. midis*, and *S. midis*/*S. sanguis*, similar to an earlier study of Zeng et al. [10]. *Streptococcus*, initially colonizing the conjunctival sac, invaded the ocular tissue causing eye infection and even endophthalmitis under low immunity, trauma, surgery, and other conditions [1]. *Streptococcus* was the most common endophthalmitis pathogen responsible for ocular trauma in children aged 3–10 years after *Staphylococcus* [12]. However, *Streptococcus* endophthalmitis has a worse treatment outcome and prognosis than *Staphylococcus* endophthalmitis [13]. In contrast to previous studies, this research not only identified the age distribution and change trend of *S. epidermidis*, *S. aureus*, and *Streptococcus* in children but also highlighted the distribution characteristics of *Streptococcus* in the conjunctival sac of children of different ages. *S. mitis* was the main bacteria in preschool children (0–6 years old). As the age increased, the proportion of *S. mitis* decreased, while the proportion of *S. pneumoniae* increased gradually. It is hypothesized that the differences in proportion may be related to factors such as immune status and hormone levels of children at different ages [14]. These findings have significant reference value for the prevention and treatment of endophthalmitis, particularly in children under 6 years old.

This study detected 35 bacterial species in male and 33 in female children, and the difference between them was not statistically significant. *S. epidermidis* accounted for the highest proportion of bacterial flora, followed by *S. aureus* and *Streptococcus*. No significant differences in the conjunctival sac flora distribution were found in normal children based on gender. However, there were differences in normal conjunctival sac flora between the eyes. The study found that only 32.16% of normal children had the same conjunctival sac flora in both eyes, indicating that most children had an asymmetric distribution of conjunctival sac flora. This may result from multiple factors. Such as the age of the children, the difference of culture condition, the error of sampling, and so on. However, we also speculate that differences in the ocular surface microenvironment may likewise play an important role. The presence of certain bacterial flora in the normal kerato-conjunctival epithelium is believed to be necessary to maintain a stable relationship with the ocular surface epithelium and stimulate the expression of IgA antibodies in the ocular surface mucosa [15]. The IgA antibodies on the ocular surface mucosa play a crucial role in neutralizing antigens and bacterial toxins, as well as in the formation of dendritic cells through interleukin-10. Therefore, it is important to take into account the individual ocular micro-environmental factors when analyzing the conjunctival sac flora. Although dry eyes have a limited effect on the distribution of conjunctival sac flora, they can increase the bacterial population [16], ultimately affecting the detection rate. Unfortunately, the study did not include individuals with dry eye signs, and a quantitative analysis of tear quality was not conducted, which may be another factor contributing to the low consistency rate of the conjunctival sac flora in both eyes.

The drug susceptibility analysis in the study found that *S. epidermidis*, *S. aureus*, and *Streptococcus* were highly sensitive to quinolones, especially to gatifloxacin and moxifloxacin, which were consistent with the previous studies [17–19]. The development of resistance to moxifloxacin is a slow process that requires a double mutation [18]. However, the widespread use of moxifloxacin in children requires close monitoring to avoid the development of resistance and to select antibiotics rationally. The study found that fusidic acid, tobramycin, and erythromycin drugs showed a high resistance rate. Fusidic acid, a narrow-spectrum and high-efficiency antibiotic, has been the first-line drug for children’s ocular inflammation in Europe, with good antimicrobial activity and low drug resistance to most *Staphylococcus* [18]. However, in recent years, there has been an increase in resistance to fusidic acid at all levels, which is attributed to the high incidence of *Haemophilus* in local children [18]. Nevertheless, this study showed an increased resistance of Gram-positive bacteria *Streptococcus* and even some *S. aureus* to fusidic acid; the *fusF* gene may be responsible for the high resistance of *Streptococcus* [20]. Additionally, we observed that *Streptococcus* had a higher resistance to fusidic acid in female children than the male children (female 89.13% > male 72.92%), and whether the expression level of the fusF gene differs between genders requires further investigation. Our findings can provide guidance for empirical drug use in remote and underprivileged areas, where drug sensitivity testing may not be feasible. The study also has implications for developing public health policies and strategies for children.

Recent studies have reported that conjunctival sac flora abnormalities are not limited to infectious eye diseases, but also observed in non-infectious eye diseases, such as allergic conjunctivitis [21] and dry eye [22]. This finding indicates that the conjunctival sac resident flora not only has a close association with infectious ophthalmopathy but also plays a crucial role in non-infectious ophthalmopathy and maintaining the normal microenvironment of the ocular surface. Therefore, understanding the normal distribution of conjunctival sac flora is crucial for comprehending the role of ocular surface microenvironment in maintaining ocular surface health and causing ocular surface diseases.

In conclusion, this study provides insights into the distribution characteristics of conjunctival sac flora in normal children aged 0–18 years and the drug susceptibility of commonly detected strains. It provides instructional guidance on ocular antibiotics selection and ocular infection treatment in children. However, the study has limitations, including a relatively small sample size and the lack of multi-center research across different regions. Further research is needed to explore the characteristics of flora drug susceptibility in children of different ages and regions.

## Data Availability

The datasets used and/or analyzed during the current study are available from the corresponding author on reasonable request.
